# Use of extended reality head-mounted displays in US health care education: a scoping review

**DOI:** 10.3389/fmed.2026.1798546

**Published:** 2026-04-14

**Authors:** Alexa Lauinger, Rachel Shrivastava, Asiya Falak, Jason Zhang, Kamryn Abraskin, Peggy Burnette, Roberto Galvez

**Affiliations:** Carle Illinois College of Medicine, University of Illinois Urbana-Champaign, Urbana, IL, United States

**Keywords:** augmented reality, extended reality, head-mounted displays, health care education, mixed reality, virtual reality

## Abstract

**Introduction:**

This scoping review examines the use of extended reality head-mounted displays (XRHMD), including virtual, augmented, and mixed reality, across medical and nursing educational settings.

**Methods:**

A comprehensive search of PubMed, CINAHL, and Scopus identified 29 U.S.-based studies that met the inclusion criteria.

**Results:**

Based on the studies examined, XR modalities were utilized across various skill and knowledge domains, including anatomy instruction, procedural rehearsal, emergency response, and surgical training. Most studies reported high learner satisfaction, citing enhanced engagement, spatial understanding, and opportunities for repeated, standardized practice. Knowledge gains were mixed, with 60% of studies showing improvement, while others demonstrated no significant advantage over traditional methods. Examination of feasibility outcomes highlighted the promise of XR for scalable, remote, and immersive instruction; however, implementation challenges were frequently reported, including technical limitations (battery life, connectivity, field of view), ergonomic concerns, and mild physiological side effects such as dizziness and visual strain.

**Discussion:**

Despite noted barriers, XR demonstrated benefits for complex spatial learning and procedural skill development, particularly when integrated into curricula with structured pre-briefing and debriefing. This review supports that XRHMD is a feasible, engaging, and educationally valuable modality, with greatest impact within blended, mastery-oriented health care education curricula where opportunities for repeated, standardized practice and three-dimensional visualization are critical for enhancing skill acquisition and spatial understanding. However, further longitudinal and standardized research is needed to inform best practices and long-term outcomes.

## 1 Introduction

Health care education continues to evolve as educators seek effective, scalable ways to help learners acquire knowledge, procedural skills, and professional behaviors. Among emerging approaches, extended reality (XR), an umbrella term covering virtual reality (VR), augmented reality (AR), and mixed reality (MR), has become a popular tool for teaching topics ranging from patient empathy and communication to anatomy and surgical procedures (1). While related, these modalities differ in how learners interact with content and the realism of the experience, which can influence the type and amount of information conveyed. VR experiences can deliver rich three-dimensional detail independent of physical setting as it immerses learners in a complete computer-generated environment. Comparatively, AR overlays digital content onto the physical world, and MR anchors virtual objects within real space to support natural interactions, which can facilitate accurate, context-aware simulations that incorporate movement and tactile feedback (1–3).

Using such XR tools has been used to enable situational learning and rehearsal tied directly to patient-specific imaging data (4). Multiple studies now suggest that XR can improve medical knowledge, skills, and learner experience. In pre-clinical cardiovascular education, a case-centered VR activity improved students’ conceptual grasp of peripheral and collateral circulation, topics difficult to observe on cadavers, illustrating XR’s value for spatially complex physiology (5). In obstetrics, an interactive XR module supported preclinical medical student learning in a problem-based learning paradigm by engaging students in anatomical and clinical exploration (6). In pediatric emergency scenarios, VR modules have been rated highly useful and associated with increased self-rated competence (3). Likewise, emergency medicine residents have indicated that VR familiarization tutorials can be helpful for reducing cognitive load before high-stakes simulations (7). For procedural training, brief, targeted VR practice added to online modules has been shown to improve objective structured technical skill scores and confidence in mannequin-based simulations (8). Smart-glasses and first-person point-of-view videos have also been positively received for reflective review and remote mentorship in nursing education (1, 9). Additionally, meta-analyses across health-professions have reported significant gains in knowledge and skill scores, shorter task times, and higher satisfaction and confidence for VR relative to traditional methods (10). Furthermore, reviews focusing on anatomy report moderate improvements in post-test scores with VR compared to conventional approaches (11, 12), while discipline-focused syntheses (e.g., orthopedics) highlight benefits for procedural rehearsal and engagement (13).

These and other studies suggest a positive future for XR’s use with health care education; however, despite promising findings, several studies have noted caution. Learners frequently cite technical friction (battery life, connectivity, field of view), ergonomics (device weight and fit), mild physiological effects (dizziness, ocular fatigue), and learning-curve burdens that can dampen educational value (2, 9). Some randomized trials in high-fidelity simulation have further reported no measurable advantage using preparatory VR sessions, underscoring the importance of sample size, curricular alignment, and adequate onboarding (14). Many reviews recommend larger, more rigorous trials, standardized outcomes, and theory-informed instructional design to clarify where XR delivers substantive educational gains versus incremental engagement (15). However, one limitation of these reviews is that they combine health care education data from multiple countries. Because learners from different countries begin their health care educational careers at different times in their life, learner expectations and prior life and educational experiences also differ (16, 17). In the U. S., health care education is characterized by longer, fragmented training, decentralized accreditation, higher costs, and future practice in a market-driven health care system (18–22). These factors affect learner expectations, engagement, and intervention outcomes differently (17), making it difficult to generalize learning activities across countries.

Building on this context, the present study provided a scoping review of XRHMD in US health care education with attention to learning outcomes (knowledge and skills), learner engagement and satisfaction, feasibility and efficiency, adverse effects across devices (e.g., HoloLens, Meta/Oculus, Magic Leap, Vuzix) and use cases (anatomy, emergency management, surgical skills, OSCEs). By mapping methodological strengths and gaps across learner levels and settings, we aim to provide actionable guidance on what types of educational activities and practices with XRHMD have shown high user satisfaction and educational benefit for curriculum designers and institutional leaders considering XR adoption.

## 2 Materials and methods

### 2.1 Study design

The Preferred Reporting Items for Scoping Reviews and Meta-Analyses extension for Scoping Reviews (PRISMA-ScR) was followed. A comprehensive search was conducted in PubMed (MEDLINE), CINAHL, and Scopus for publications related to XR head-mounted displays (XRHMD) and health-professions education. Search strategies were developed with a medical/biomedical librarian. The core search string combined terms for XR (virtual/augmented/mixed reality), head-mounted displays (e.g., smart glasses, HoloLens, Oculus, Magic Leap), and education/training in nursing and medicine (Table 1; Supplementary Appendix 1).

**Table 1:** Library search concepts.

Search Concepts, MeSH Terms, and Keywords
1 Augmented reality [MeSH] = augmented reality; virtual reality [keywords] = extended reality; virtual reality; augmented reality; mixed reality
2 XR devices [MeSH] = smart glasses [keywords] = head-mounted display; virtual reality headset; optical head-mounted display; smart glasses; smartglasses; google glasses; hololens; oculus rift; magic leap
3 Medical or nursing students [MeSH] = students, medical; students, nursing [keywords] = medical students; nursing students; interns; residents
4 Medical or nursing education [MeSH] = education, medical; nursing education [keywords] = medical education; nursing education

### 2.2 Scoping review protocol

All of the citations were imported into a reference-management software and duplicates were removed. The first 50 articles were screened by all six reviewers to establish uniform procedure. Fleiss’ kappa (*κ*) and a pairwise percent agreement was used to quantify agreement among the reviewers for each of the five binary screening fields (study/publication type; population; XR intervention; head-mounted device; and location), with bootstrap 95% confidence intervals (2,000 resamples). Inconsistencies in this initial screening were resolved through group discussions and consensus. The remaining articles were screened in two stages. In the first stage, two reviewers independently screened each article using the title/abstract for eligibility using a standardized exclusion code: ST (study type), NP (not study population), NX (no XR technology), and NU (non-US context). Articles were excluded if they were narrative reviews or gray literature; if participants were outside medicine, nursing, PA, pharmacy, dentistry, or veterinary programs; if the intervention lacked immersive XR; or if the educational context occurred outside the United States. As indicated above U. S. health care education is characterized by multiple factors that could differentially shape learner experiences and impact educational expectations. All articles, including the 50 initial articles, that did not meet the exclusion criteria or if the exclusion criteria could not be determined from the title or abstract were moved to the second stage screening process. In this stage the entire article was used with the exclusion criteria outlined above. Screening discrepancies in stage one and two were resolved by a third reviewer.

### 2.3 Data extraction and analysis

Following screening, each article underwent data extraction by two independent reviewers. Results from these reviewers were combined with discrepancies resolved by a third reviewer prior to inclusion. Features captured included modality (VR/AR/MR), device, intervention, setting, participant population, learning measures, participant perspectives, costs, barriers to adoption, and adverse effects. Given heterogeneous protocols and outcomes, results were synthesized qualitatively without meta-analysis.

## 3 Results

### 3.1 Study selection and characteristics

Our initial search resulted in 609 citations. From those 173 duplicates were removed leaving 436 citations. To assess interrater reliability, all six reviewers screened the first 50 references using the title and abstract. Interrater reliability was then evaluated with Fleiss’ kappa (*κ*) and a pairwise percent agreement (PPA) for the five binary screening fields (study/publication type κ = 0.304 (95% CI − 0.038 to 0.561), PPA = 89.1%; population κ = 0.720 (95% CI 0.481 to 0.898), PPA = 85.1; XR intervention κ = 0.122 (95% CI − 0.013 to 0.193), PPA = 98.1%; head-mounted device κ = 0.172 (95% CI 0.111 to 0.202), PPA = 88.4%; location κ = 0.832 (95% CI − 0.143 to 1.000), PPA = 94.1%). Confidence intervals were estimated via bootstrap resampling (2,000 iterations). Any inconsistencies in this initial screening were resolved through group discussion and consensus.

**Figure 1:**
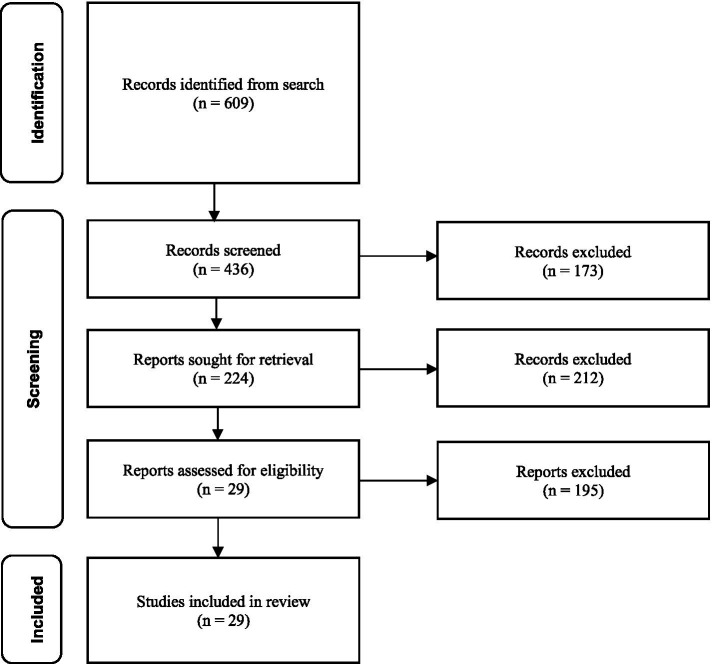
Citation search and screening flowchart.

**Figure 2:**
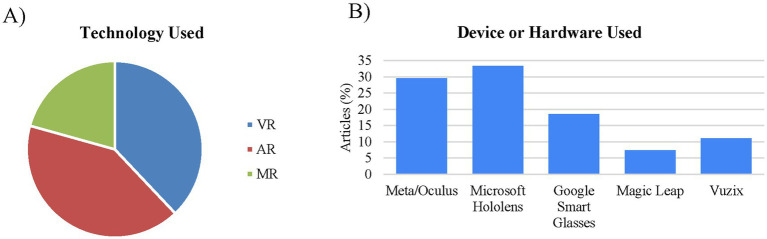
Type of device and technology primarily used for XR health care education. **(A)** Technology used: most of the studies examined (41%; *n* = 12) used augmented reality (AR) while 38% (*n* = 11) used virtual; reality (VR), and the remaining 21% (*n* = 6) used mixed reality (MR). **(B)** Device or hardware used: The primary device used in the studies examined was the Microsoft HoloLens (33%; *n* = 9).

In the first stage of the screening process that included the above mentioned 50 citations and focused on the title and abstract, 212 articles were removed based on the exclusion criteria leaving 224 citations. In the second stage of the screening process an additional 195 articles were removed based on the exclusion criteria leaving 29 citations (Figure 1). Data extraction was then conducted on these 29 studies (Table 2). Of these, 41% (*n* = 12) used AR, 38% (*n* = 11) used VR, and 21% (*n* = 6) used MR. From the studies that indicated the device used (*n* = 27), the most common were Microsoft HoloLens (33%; *n* = 9), Meta/Oculus (30%; *n* = 8), and Google smart glasses (19%; *n* = 5) (Figure 2).

**Table 2:** Studies included in the analysis.

Study	Goal	Study design	Participants and setting
Armstrong et al. (2014) (28)	Knowledge (Surgical procedure)	*Technology* VR *Device* Google Glass *Intervention* The surgical procedure was cast to the phone of a resident that allowed them to view the surgery and a remote consultant highlighted important aspects of the anatomy and technique.	*n* = 1 (junior residents) Location: Operating room
Dickey et al. (2016) (31)	Knowledge (Surgical procedure)	*Technology* VR *Device* Google Glass *Intervention* Participants could view the surgical procedure to see important aspects of the anatomy and technique.	*n* = 20 (urology residents) Location: Operating room
Vaughn et al. (2016) (44)	Knowledges (Patient management)	*Technology* AR *Device* Google Glass *Intervention* A video of a patient suffering from acute respiratory distress was displayed alongside a high-fidelity manikin simulation.	n = 12 (nursing students) Location: Classroom
Stepan et al. (2017) (41)	Knowledge (Neuroanatomy)	*Technology* VR *Device* Oculus Rift *Intervention* Students used either VR or a textbook to study the anatomy of the brain and then were quizzed on the material.	*n* = 66 (medical students) Location: Classroom
Dang et al. (2018) (30)	Knowledge (Patient management)	*Technology* VR *Device* Oculus Rift *Intervention* A simulation was completed in VR or with a televised education session.	*n* = 58 (nursing students) Location: Classroom
Farra et al. (2018) (52)	Knowledge (Patient management)	*Technology* VR *Device* Oculus Rift *Intervention* Students went through a disaster-based simulation using traditional methods or with VR.	*n* = 32 (nursing students) Location: Classroom
Dickerson et al. (2019) (54)	Skill (Surgical fracture reduction)	*Technology* VR *Device* Google Glass *Intervention* Students completed a simulated task for fracture reduction with one group receiving video coaching after the session.	*n* = 42 (orthopedic surgery residents) Location: Simulated operating room
Kardong-Edgren et al. (2019) (36)	Skill (Sterile placement of a urinary catheter)	*Technology* AR *Device* Oculus Rift *Intervention* Students used a headset to view a hospital setting with a simulation manikin then they had to place a urinary catheter.	*n* = 31 (nursing students) Location: Classroom
Rojas-Muñoz et al. (2020) (49)	Skill (Surgical abdominal incisions)	*Technology* AR *Device* HoloLens *Intervention* Participants completed anatomical marking and an abdominal incision in a simulation with two groups using AR technology or an instruction over telephone.	*n* = 20 (medical students) Location: Simulated operating room
Stojanovska et al. (2019) (42)	Knowledge (Anatomy)	*Technology* MR *Device* HoloLens *Intervention* Students studied upper limb anatomy using the MR technology or traditional cadaver dissection.	*n* = 64 (medical students) Location: Anatomy lab
Breitkreuz et al. (2021) (29)	Skill (Catheter placement)	*Technology* VR *Device* Samsung Gear *Intervention* A VR simulation game was created to allow students to practice the placement of urinary catheters.	*n* = 300 (nursing students) Location: Classroom
Robinson et al. (2020) (37)	Knowledge (Anatomy)	*Technology* MR *Device* HoloLens *Intervention* Students used HoloLens MR models and embedded histology images to complete a faculty-led respiratory anatomy session.	*n* = 10 (medical students) Location: Anatomy lab
Rojas-Muñoz et al. (2020) (50)	Skill (Leg fasciotomy)	*Technology* AR *Device* HoloLens *Intervention* Trainees performed leg fasciotomies on cadaveric specimens under telementoring or reviewing the procedure beforehand.	*n* = 20 (residents and medical students) Location: Simulated operating room
Wunder et al. (2020) (39)	Skill (Tracheostomy)	*Technology* MR *Device* Magic Leap One *Intervention* Fire scenario with virtual smoke fire and water during simulated tracheostomy.	*n* = 34 (nursing anesthetist students) Location: Simulated operating room
Abulfaraj et al. (2021) (55)	Knowledge (Patient management)	*Technology* VR *Device* Oculus Rift *Intervention* Participants completed a VR simulation of a pediatric status epilepticus case compared to a high-fidelity mannikin.	*n* = 42 (residents) Location: Classroom
Frederick and Gelderen (2021) (48)	Knowledge (Patient care)	*Technology* AR *Device* Vuzix M100/M300 *Intervention* Participants watched a simulation and filled out surveys regarding the use of AR for learning.	*n* = 50 (nursing student) Location: Classroom
Yanni et al. (2021) (47)	Skill (Screw placement)	*Technology* AR *Device* Magic Leap One *Intervention* Participants used AR to place spinal fusion screws	*n* = 6 (residents and medical students) Location: Simulated operating room
Collier et al. (2023) (51)	Knowledge (Navigating cultural boundaries)	*Technology* VR *Device* Google Cardboard *Intervention* VR video about a topic that spans cultural boundaries.	n = NR (nursing students) Location: Classroom
Donovan (2023) (32)	Knowledge (Patient management)	*Technology* AR *Device* HoloLens *Intervention* A patient simulation using AR technology for management and decision making.	*n* = 18 (medical students) Location: Classroom
Gandsas et al. (2023) (40)	Knowledge (Procedural knowledge)	*Technology* VR *Device* Oculus Quest 2 *Intervention* Participants viewed a laparoscopic sleeve gastrectomy remotely using VR.	*n* = 10 (Surgery residents) Location: Operating room
Kim et al. (2023) (33)	Skill (IV insertion)	*Technology* MR *Device* HoloLens 2 *Intervention* Participants used the device with haptic gloves to practice insertions on various vein presentations.	*n* = 20 (nursing and medical students) Location: Classroom
Rossitto et al. (2023) (38)	Skill (EVD placement)	*Technology* VR *Device* Oculus Rift *Intervention* Participants completed repeated VR EVD placement trials.	n = 29 (residents and medical students) Location: Simulated operating room
Sridhar et al. (2023) (46)	Skill (Vaginal delivery)	*Technology* AR *Device* Vuzix M400 *Intervention* A birthing simulator and smart glasses were used to livestream the simulated delivery, and a previously published checklist was used to show the steps for conducting routine vaginal delivery.	*n* = 62 (medical students) Location: Delivery room
Swan et al. (2023) (27)	Knowledge (Social determinants of health)	*Technology* VR *Device* Oculus Quest 2 *Intervention* Participants viewed a VR simulation to educate them about social determinants of health.	*n* = 10 (nursing students) Location: Classroom
Erickson et al. (2024) (45)	Skill (Guidewire placement)	*Technology* MR *Device* HoloLens *Intervention* A patient specific preprocedural MR training program for 10–15 min followed by glenoid guidewire placement with MR holographic assistance was tested compared to freehand guidewire placement.	*n* = NR (residents) Location: Classroom
Kim et al. (2024) (35)	Skill (IV insertion)	*Technology* MR *Device* HoloLens *Intervention* They utilized exoskeleton haptic gloves for left hand interaction with the virtual patient hand and a modified stylus haptic device to perform IV needle insertion.	*n* = 31 (nursing students) Location: Classroom
Lovett et al. (2024) (43)	Skill (Suturing)	*Technology* AR *Device* HoloLens *Intervention* Students used an AR suture guidance application to assist the independent practice of suturing.	*n* = 30 (medical students) Location: Classroom
Meliagros et al. (2024) (53)	Skill (Lumbar puncture procedure)	*Technology* MR *Device* Meta Oculus *Intervention* Students watched the virtual first-person teaching via a virtual reality headset while mimicking hand movements on a lumbar puncture mannikin.	*n* = 58 (medical students) Location: Classroom
Shepard et al. (2025) (34)	Skill (Ultrasound-guided percutaneous nephrolithotomy)	*Technology* AR *Device* Vuzix M400 *Intervention* Students completed simulated nephrolithotomy with smart glasses and proctored by experts that gave instructions through the procedure.	*n* = 12 (urology residents) Location: Simulated operating room

**Figure 3:**
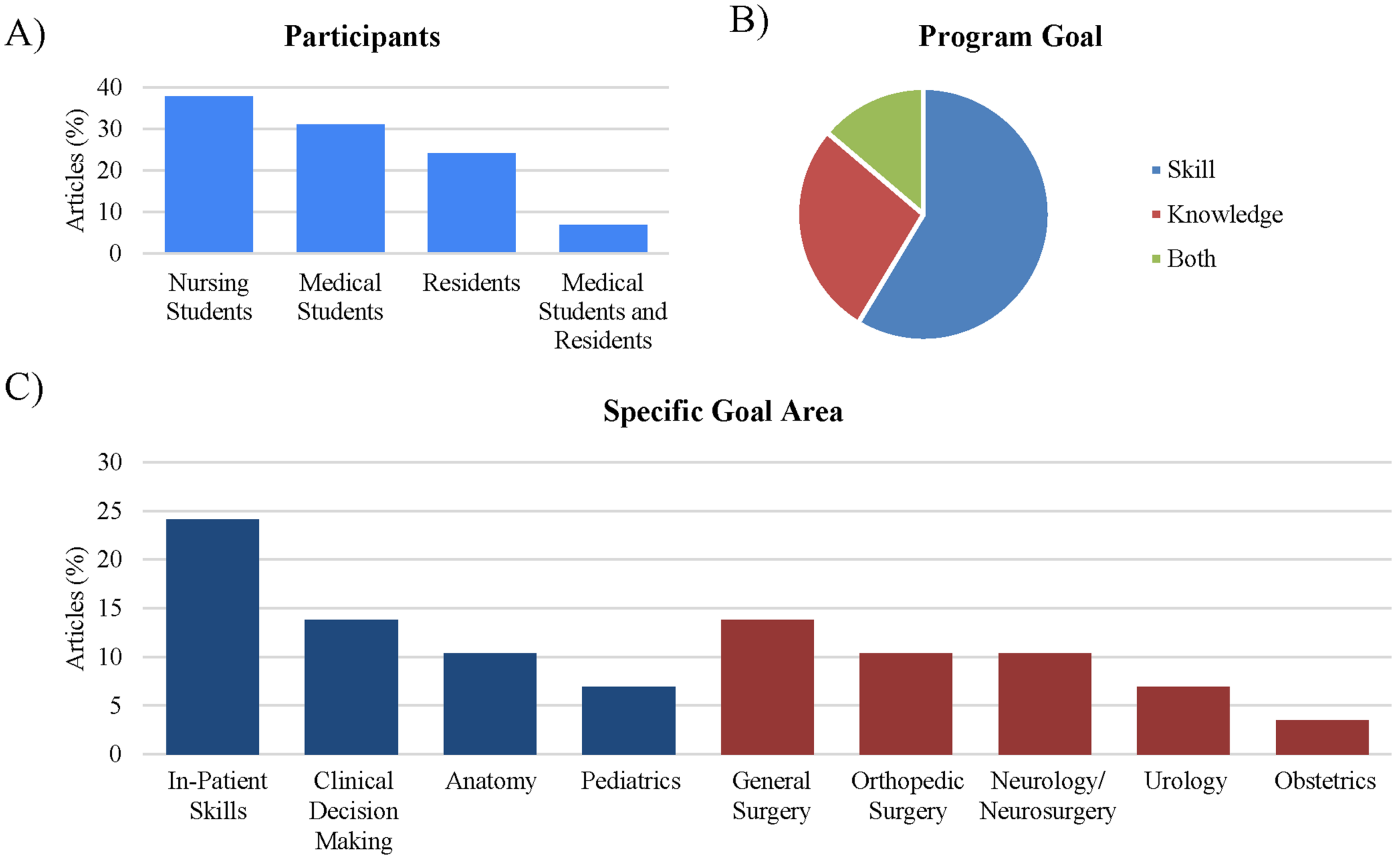
Breakdown of the study participants and educational goals. **(A)** Participants: nursing students were the primary participants for the studies examined (38%; *n* = 11). **(B)** Program goal: XR use with health care education primarily focuses on skills (59%; *n* = 17) or skills with knowledge (14%; *n* = 4). Only 28% (*n* = 8) of the studies assessed only knowledge. **(C)** Specific goal area: when looking at XR educational goals, most of the studies looked at surgical skills (red bars, 45%; *n* = 13), while 24% (*n* = 7) of the studies focused on in-patient skills.

Participants were predominantly nursing students (38%; *n* = 11), followed by medical students (31%; *n* = 9) and residents (24%; n = 7) with two studies including both medical students and residents (7%). None of the studies meeting the inclusion criteria involved students from physician assistant, pharmacy, dentistry, or veterinary programs. Looking at the study goal, the majority assessed skills with 59% (*n* = 17) assessing skills, 28% assessing knowledge (*n* = 8), and 14% assessing both knowledge and skills (*n* = 4). Looking more closely at specific goal area it was determined that 24% (*n* = 7) tested general adult inpatient skills (e.g., IV insertion, catheter placement, acute respiratory assessment, fire protocols, decontamination); 14% (*n* = 4) examined non-surgical decision-making and barrier recognition; 7% (*n* = 2) covered pediatric scenarios (anaphylaxis, status epilepticus); and 10% (*n* = 3) focused on cadaver-based anatomy classes (respiratory, musculoskeletal, neurological). The remaining studies addressed surgical skills: 14% (*n* = 4) general surgery (suturing, abdominal marking/incisions, laparoscopic sleeve gastrectomy); 10% (*n* = 3) orthopedic surgery (glenoid guidewire placement, ORIF, leg fasciotomy); 10% (*n* = 3) neurology/neurosurgery (lumbar puncture, EVD placement, pedicle screws); 7% (*n* = 2) urology (penile prosthesis, percutaneous nephrolithotomy); and 3% (*n* = 1) obstetrics (vaginal delivery training) (Figure 3).

### 3.2 Synthesized findings

**Figure 4:**
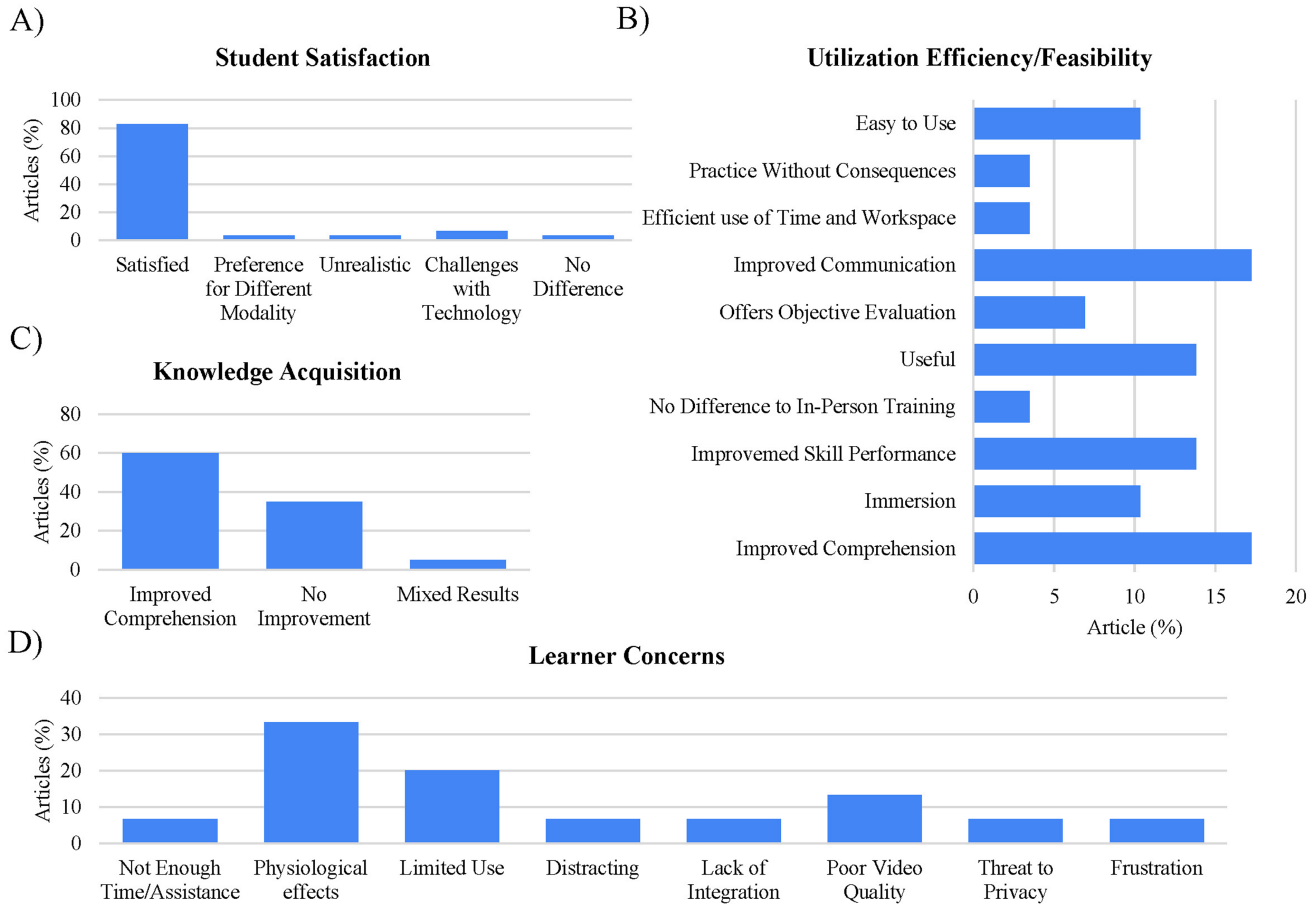
Student satisfaction using XR with health care education. **(A)** Student satisfaction: students were overwhelmingly satisfied with the use of XR in educational activities (83%; *n* = 24). **(B)** Utilization efficacy/feasibility: some of the most reported benefits include improved communication (17%; *n* = 5). **(C)** Knowledge acquisition: from the articles that examined knowledge acquisition (*n* = 20) 60% (*n* = 12) reported improved comprehension. However, 35% (*n* = 7) reported no improvement. **(D)** Learner concerns: The most prominent reported learner concern from the studies that reported learner concerns (*n* = 15) included physiological effect (33%; *n* = 5), limited use (20%; *n* = 3), and video quality (13%; *n* = 2).

Exploring student satisfaction, most studies (83%; *n* = 24) indicated favorable experiences. Participants cited improved comprehension (17%; *n* = 5), communication (17%; *n* = 5), skill performance (14%; *n* = 4), and described XR as generally useful (14%; *n* = 4). Among studies evaluating knowledge acquisition (*n* = 20), 60% (*n* = 12) reported improvements, though 35% (*n* = 7) found no advantage versus conventional educational methods, and 5% (*n* = 1) noted mixed results. Interestingly, 31% (*n* = 9) of the studies examined (*n* = 29) did not assess learning outcomes (Figure 4). Of the 15 studies that reported learner concerns, physiological effects such as dizziness and ocular fatigue were reported in 33% (*n* = 5) of the articles with user limitations such as left-handed use, wearing glasses, peripheral vision, learning curve, discomfort reported in 20% (*n* = 3) or the articles.

**Figure 5:**
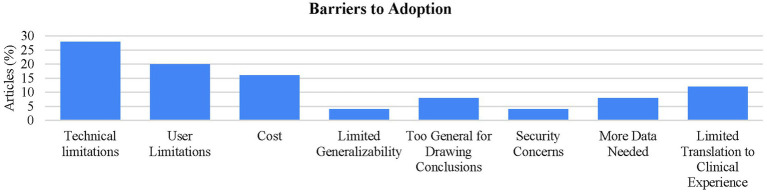
Examination of student concerns and barriers for adoption. Barriers to adoption: from the studies that reported barriers to adoption (*n* = 25), technical limitations were found to be the largest barrier for adoption (28%; *n* = 7).

Interestingly, 25 of the studies also noted several barriers to implementation. Of these barriers, technical limitations (battery life, connectivity, hardware constraints, latency) were the most prevalent (28%; *n* = 7). Although also reported in the analysis of student satisfaction (Figure 4D), user limitations (left-handed use, wearing glasses, peripheral vision, learning curve, discomfort) were included in the barriers to implementation analysis as many studies reported it as an implementation barrier (20%; *n* = 3). In addition, concerns around implementation cost were reported in 16% (*n* = 4) of these studies (Figure 5). Of the articles that discussed implementation costs (*n* = 8), 50% (*n* = 4) indicated concerns with the expense as reported above, while the other 50% (*n* = 4) indicated that using XR was affordable or cost effective.

## 4 Discussion

### 4.1 Summary of findings

As XR becomes more prominent in health care education, understanding protocol choices and their effects on learner outcomes is essential for thoughtful adoption (4, 15). While XRHMD presents a unique opportunity to fully immerse trainees in the educational session, they are also associated with greater risks of adverse physiological effects, usually nausea, visual strain, and physical discomfort (1, 5, 10, 23). Furthermore, while many studies reported higher enjoyment and engagement, benefits with knowledge acquisition were mixed when compared to traditional methods, patterns consistent with prior reviews of XR in health-professions education across multiple countries (10, 15, 24). Additionally, it should be noted that nearly one-third (31%) of the studies reviewed did not evaluate learning outcomes. This gap in the literature is significant, particularly as many educational institutions continue to consider the efficacy of this technology for enhancing academic achievement.

When integrating XRHMD into health care education, a few common strengths in the studies examined included engagement, realism, and ease of use. Other benefits that were briefly touched on in this review and have been suggested in other research are that XR systems allow for more frequent repetition of information and for more uniform teaching methods across groups (11). The current study along with others have found mixed results with knowledge acquisition (14, 15). Collectively these studies suggest that institutions should consider prioritizing XRHMD deployment for skills development (e.g., IV placement, suturing, laparoscopic tasks) and complex spatial reasoning (e.g., anatomy, surgical rehearsal), where evidence shows clearer benefits (8, 11, 12).

XRHMD benefits also appear to scale with task complexity and the availability of structured practice. These findings suggest that early exposure in preclinical training would have maximal long-term benefits (8, 13, 24). Many studies have further suggested that XR be situated within blended, mastery-oriented sequences rather than deployed as a stand-alone novelty. Deliberate-practice cycles that interweave cognitive scaffolds (short concept videos, checklists), XR rehearsal (procedural steps, spatial orientation), and debriefing (reflective prompts, feedback) are promising educational uses. For anatomy or imaging-heavy topics, pairing XR with patient-specific or near-isotropic 3D datasets can strengthen clinical relevance for procedural skills. Importantly, some studies have suggested that less-immersive modes may outperform fully immersive ones for declarative knowledge targets, reinforcing a “right tool for the job” principle (4, 15, 24).

Although this study reported several positives with XRHMD with health care education, there are still many reported human-factor and implementation constraints. Adverse effects such as dizziness, ocular fatigue, and discomfort, although typically mild, were commonly reported and can greatly hinder focusing during learning experiences (14, 23). In addition, practical barriers, including headset ergonomics for eyeglass wearers, field-of-view limitations, battery life, and network reliability, were reported in some of the studies examined and can also erode learning time and instructor outcomes. Some of these technical and human-factors constraints (battery life, latency, field of view, headset weight, eyewear compatibility) can be anticipated and mitigated through integration with existing clinical and classroom infrastructure, device trials, onboarding, and iterative instructional design (2, 23, 25, 26).

### 4.2 Limitations

While this review explores the unique implications of XRHMD with health care education, there are several limitations. The selection criteria for this review excludes any articles published outside of the United States or any articles that did not have the full text available. Although this selection improves standardization, it may also cause studies with results relevant to the useability of XRHMD for the health care education to be excluded. Additionally, these studies reported a wide range of outcomes based on different surveys and measurements and therefore could not be compared through a meta-analysis. Future work should attempt to standardize reporting variables and include longitudinal designs linking XRHMD exposure to downstream performance (e.g., OSCEs, workplace-based assessments) and patient-centered outcomes (15, 26). Additionally, future work is needed to explore how long-term application of XRHMD into the curricula can impact non-immediate skill retention and comprehensive exam scores.

## 5 Conclusion

With the growing use of XR in health care education, it is essential to explore the benefits and limitations of these technologies within different health care curricula. This scoping review focused on research using XRHMD in a range of health care educational settings including simulated operating rooms and the anatomy classroom. Although not assessed in all of the studies examined, XRHMD demonstrated educational value when deployed selectively for educational activities that benefit from immersive, spatial, and procedural learning rather than stand-alone instructional replacements. XRHMDs demonstrate educational value for procedural skill development, surgical rehearsal, emergency response training, and spatially complex content such as anatomy and imaging interpretation, where opportunities for repeated, standardized practice and three-dimensional visualization are critical. In contrast, gains in declarative knowledge are mixed, indicating that XRHMDs should supplement rather than replace conventional instructional methods for factual learning. Studies that demonstrated successful implementation were also consistently associated with a curricular structure that included pre-briefing, guided XR practice before engaging in the educational activity, and post-experience debriefing. Educators should also anticipate and develop a plan to mitigate human-factors and technical constraints, such as device ergonomics, motion sensitivity, battery life, and connectivity, to avoid disrupting instructional flow. Recognizing these implementation guidelines, the findings support the use of XRHMD within blended, mastery-oriented health care education curricula to enhance skill acquisition and spatial understanding while minimizing unnecessary cognitive and logistical burden.

## Data Availability

The original contributions presented in the study are included in the article/Supplementary material, further inquiries can be directed to the corresponding author.
