# Supplementary material for: Use of extended reality head-mounted displays in US health care education: a scoping review
Source: Front Med (Lausanne). 2026 Apr 14;13:1798546. doi: 10.3389/fmed.2026.1798546 (PMC13121061; doi:10.3389/fmed.2026.1798546)
Supplement: Supplementary file 1 [file Data_Sheet_1.PDF]

Search Strategy Record

Research Question:

What is the nature, extent, and impact of immersive extended reality technologies in medical, nursing, physician assistant, and pharmacy education programs, both undergraduate and graduate, in the US?

Source__PubMed______________________________________

Date Searched___April 29, 2025____________________________

SEARCH#	SEARCH STRING	NOTES
1	(Augmented reality[mesh] **OR** virtual reality[mesh])	[*concept* 1] 10,000
2	(extended reality [tiab] **OR** virtual reality[tiab] **OR** augmented reality[tiab] **OR** mixed reality[tiab])	[*concept 1*] 28,601
3	(simulation training[mesh] **OR** simulation[tiab])	[*concept 1]* 322,987
4	1 OR 2 OR 3	348,307
5	(Smart glasses[mesh] **OR**<br><br>"head-mounted display" **OR** “virtual reality headset” **OR** “optical head-mounted display” **OR** “smart glasses” **OR** “google glasses” **OR** hololens **OR** “oculus rift” **OR** "magic leap")	[*concept 2*] 2,539
6	4 AND 5	1,917
7	(Students, medical[mesh] **OR** students, nursing[mesh])	[*concept 3*] 80,936
8	(Medical students[tiab] **OR** nursing students[tiab] **OR** interns **OR** residents)	[*concept 3*] 697,777
9	7 OR 8	731,838
10	(Education, medical[mesh] **OR** education, nursing[mesh])	[*concept 4*] 284,299
11	(nursing[tiab] OR medicine[tiab] OR medical[tiab]) AND (education[tiab] OR training[tiab] OR instruction[tiab])	[*concept 4]* 344,945

12	10 OR 11	526,304
13	9 OR 12	1,094,177
15	6 AND 13	248
16	15 years	237
17	English	235

Limits/Restrictions/Filters

English

15 years

Source__CINAHL____

Date Searched____May _5, 2025_____________________________

SEARCH#	SEARCH STRING	NOTES
1	("extended reality" OR "virtual reality" OR "augmented reality" OR "mixed reality") [IN title/abstract]	8,695
2	(MH "Virtual Reality") OR (MH "Augmented Reality)	7,268
3	((MH "Simulations") OR (MH "Augmented Reality"))	20,619
4	1 OR 2 OR 3	31,272
5	(MH "Smart Glasses") OR (("head-mounted display" OR “virtual reality headset” OR “optical head-mounted display” OR “smart glasses” OR “google glasses” OR hololens OR “oculus rift” OR "magic leap"))	552
6	4 AND 5	396
7	((MH "Students, Medical") OR (MH "Students, Nursing")) OR XB ("medical students" OR "nursing students")	78,040
8	(MH "Education, Medical") OR (MH "Education, Nursing")	87,306
9	XB (education OR training OR instruction) AND XB (nursing OR medicine OR medical) {IN title/abstract]	158,003

10	8 OR 9	214,266
11	7 OR 10	252,244
12	6 AND 11	60
13	15 years	59
15	English	55

Source__Scopus

Date Searched_ May 7, 2025________________________________

SEARCH#	SEARCH STRING	NOTES
1	("extended reality" OR "virtual reality" OR "augmented reality" OR "mixed reality") [IN title/abstract/keywords]	243,237
2	(("head-mounted display" OR “virtual reality headset” OR “optical head-mounted display” OR “smart glasses” OR “google glasses” OR hololens OR “oculus rift” OR "magic leap")) [IN title/abstract/keywords]	17,337
3	1 AND 2	12,658
4	("medical students" OR "nursing students") [title/abstract/keywords]	159,968
5	XB (education OR training OR instruction) AND XB (nursing OR medicine OR medical) [title/abstract/keywords]	1,087,642
6	4 OR 5	1,129,045
7	3 AND 6	688
8	15 years	651
9	English	636
10	Limit to Article	319

Total databases results = 609

Deduplication = 436
